# Tablet-Based Functional MRI of the Trail Making Test: Effect of Tablet Interaction Mode

**DOI:** 10.3389/fnhum.2017.00496

**Published:** 2017-10-24

**Authors:** Mahta Karimpoor, Nathan W. Churchill, Fred Tam, Corinne E. Fischer, Tom A. Schweizer, Simon J. Graham

**Affiliations:** ^1^Department of Medical Biophysics, Sunnybrook Research Institute, University of Toronto, Toronto, ON, Canada; ^2^Neurosurgery Department, Keenan Research Centre of the Li Ka Shing Knowledge Institute, St. Michael's Hospital, Toronto, ON, Canada; ^3^Geriatric Psychiatry, Psychiatry Department, St. Michael's Hospital, Toronto, ON, Canada

**Keywords:** trail making test, fMRI, neuropsychological tests, pen-and-paper test, executive function, visual feedback of hand position

## Abstract

The Trail Making Test (TMT) is widely used for assessing executive function, frontal lobe abilities, and visual motor skills. Part A of this pen-and-paper test (TMT-A) involves linking numbers randomly distributed in space, in ascending order. Part B (TMT-B) alternates between linking numbers and letters. TMT-B is more demanding than TMT-A, but the mental processing that supports the performance of this test remains incompletely understood. Functional MRI (fMRI) may help to clarify the relationship between TMT performance and brain activity, but providing an environment that supports real-world pen-and-paper interactions during fMRI is challenging. Previously, an fMRI-compatible tablet system was developed for writing and drawing with two modes of interaction: the original cursor-based, proprioceptive approach, and a new mode involving augmented reality to provide visual feedback of hand position (VFHP) for enhanced user interaction. This study characterizes the use of the tablet during fMRI of young healthy adults (*n* = 22), with half of the subjects performing TMT with VFHP and the other half performing TMT without VFHP. Activation maps for both TMT-A and TMT-B performance showed considerable overlap between the two tablet modes, and no statistically differences in brain activity were detected when contrasting TMT-B vs. TMT-A for the two tablet modes. Behavioral results also showed no statistically different interaction effects for TMT-B vs. TMT-A for the two tablet modes. Tablet-based TMT scores showed reasonable convergent validity with those obtained by administering the standard pen-and-paper TMT to the same subjects. Overall, the results suggest that despite the slightly different mechanisms involved for the two modes of tablet interaction, both are suitable for use in fMRI studies involving TMT performance. This study provides information for using tablet-based TMT methods appropriately in future fMRI studies involving patients and healthy individuals.

## Introduction

Since its development by U.S. Army psychologists in the latter stages of the second world war, the Trail Making Test (TMT) has become one of the most widely used neuropsychological (NP) tools for assessing brain dysfunction in diverse patient populations (Halstead, 1947; Morris et al., 1987; Dikmen et al., 1990; Buchanan et al., 1994; Ashendorf et al., 2008; McKhann, 2011). The TMT is normally administered in two sub-components that are known as TMT-A and TMT-B. In TMT-A, the patient is presented with encircled numbers from 1 to 25 randomly distributed on a sheet of paper, and they are instructed to link the numbers in ascending order (i.e., 1-2-3…) using a pen or pencil. In TMT-B, a second sheet includes both encircled numbers and letters that the patient must link in alternating ascending order (i.e., 1-A-2-B-…). Task performance in each part is typically quantified by measuring the completion time, with TMT-B taking longer to complete. The difference between the completion times for TMT-A and TMT-B (i.e., “B-A”) is frequently used to remove the speed element from the test evaluation, and ratio scores such as B/A (Corrigan and Hinkeldey, 1987) and (B-A)/A (Stuss et al., 2001; Bowie and Harvey, 2006) have also been employed to assess a variety of cognitive impairments (Bowie and Harvey, 2006).

As it requires elements of visuomotor tracking, scanning, divided attention and cognitive flexibility (Kortte et al., 2002), the TMT is commonly used as a clinical measure of executive functions—cognitive processes such as problem solving, attentional control, monitoring behavior, and mental ability to switch between two different concepts simultaneously—which are strongly associated with the frontal lobes (Reitan, 1971; Stuss et al., 2001; Lezak et al., 2004; Mitrushina et al., 2005; Strauss et al., 2006; Sánchez-Cubillo et al., 2009). However, factors which affect TMT performance continue to be investigated and clarified. For example, the increase in completion time required for TMT-B compared to TMT-A has been attributed to increased demands in motor speed, visual search, and executive function (Stuss et al., 2001; Sánchez-Cubillo et al., 2009); longer trail length (Rossini and Karl, 1994) and increased visual interference in the tracing path (Gaudino et al., 1995). It is also well-appreciated that patients may be impaired or unimpaired on the TMT for diverse reasons according to the specific disease state and the associated pattern of regional brain dysfunction (Levine et al., 1998; Stuss et al., 2001).

Furthermore, it is overly simplistic to consider the TMT solely as a test of executive function. In reality, the subject must receive sensory inputs and then process them executively to provide the appropriate motor responses for successful TMT performance. A complicated network of brain regions is implicated, which must be slightly different between TMT-A and TMT-B, and the amplitude and extent of activity across the brain has not been studied yet in sufficient detail for both parts of the test. Traditionally, the brain regions that support TMT performance have been investigated through lesion studies (Levine et al., 1998; Stuss et al., 2001). Lesion studies have fundamental weaknesses, however, such as cases where lesions are located at different sites but the patients have similar test performance; or cases where lesions are located in similar sites with the same pathology, but patients have different test performance (Lezak et al., 2004; Hebben and Milberg, 2009). Therefore, efforts to inform appropriate usage of the TMT and understanding underlying mental processes involved during TMT performance have started to include simultaneous measurement of behavior and brain activity using functional neuroimaging (Moll et al., 2002; Zakzanis et al., 2005; Allen et al., 2011). Such approaches overcome the known limitations of lesion studies for localizing brain functions (Friston and Price, 2001). Of the various modalities available, functional magnetic resonance imaging fMRI is recognized as an important tool (Lezak et al., 2004; Hebben and Milberg, 2009) that provides a safe, non-invasive method to probe neuronal activity indirectly through the associated localized changes in blood oxygenation, flow, and volume (Ogawa et al., 1990, 1992).

Performing complex pen-and-paper tests during fMRI involves engineering challenges, however. A robust design is required to enable behavioral performance during fMRI that is very similar to performance of the TMT under normal office testing conditions. Although attempts have been made to determine the brain activity of the TMT using simplified or modified tasks (e.g., Moll et al., 2002) involving common fMRI-compatible devices, such as a push-button fiber optic response pad (Allen et al., 2011), these approaches may report biased maps of brain activity as the task requirements and response mode have been substantially altered in relation to the “real-world” version of the TMT. For example, early work by Moll et al. (2002) reported that the executive functioning component of the TMT activated the left dorsolateral prefrontal cortex (DLPFC), supplementary motor area, and the cingulate sulcus. However, Allen et al. (2011) had slightly different findings that included activation of ventral and dorsal visual pathways and the medial pre-supplementary motor areas in addition to the left DLPFC. These differences in reported activation maps have spurred other investigators to develop and adopt specialized devices that enable realistic writing and drawing behavior during fMRI. For example, a fiber-optic MRI compatible writing device was developed to map brain activity associated with TMT performance (Zakzanis et al., 2005). The study reported involvement of the left DLPFC during TMT-B, as well as the precentral gyrus, cingulate gyrus, and medial frontal gyrus, suggesting the importance of both motor control and cognitive flexibility for this part of the test. To overcome certain technical limitations of the fiber-optic device, a computerized fMRI-compatible tablet system (consisting of a touch-sensitive screen that could be operated by finger or using a stylus) was subsequently designed and validated (Tam et al., 2011). To date, the prototype has been used in multiple fMRI studies of healthy adults, for example to investigate aspects of bimanual co-ordination when tracing lines (Callaert et al., 2011), to investigate creative processes involved in drawing (Ellamil et al., 2012), to improve fMRI pre-processing pipeline optimization and data analysis methods using a simplified version of the TMT (Churchill et al., 2015), and to study the sustained attention to response task (SART) in healthy adults (Churchill et al., 2012b).

The fMRI-compatible tablet was originally developed with a cursor-based approach for user interactions with a stylus. With the stylus appropriately positioned on the tablet surface, the user depresses a sensor at the stylus tip to trigger recording of writing and drawing movements on a computer display for viewing purposes. Because the user lies supine in the magnet bore with the tablet mounted over their torso, they are unable to view their hand, the stylus and the tablet surface while making such interactions. Instead, they must rely on feedback from the cursor and their sense of proprioception to place the stylus tip at required locations on the tablet surface. Although healthy adults learn to make tablet responses readily in this manner, the cursor-based interaction mode was recognized as a factor that (a) potentially increased task demands in comparison to real-world stylus interactions; and (b) could be an important potential confound in interpreting brain maps obtained from tablet-based fMRI, particularly for patients with brain impairments and older/younger cohorts which may learn at different rates. An additional interaction mode was subsequently developed to address these concerns. A video camera and augmented reality display was included to provide visual feedback of hand position VFHP, demonstrating improved writing performance in relation to cursor-based interactions in young healthy adults (Karimpoor et al., 2015).

Although promising initial findings were obtained for tablet interactions with VFHP, the supporting fMRI data were rather limited and were collected specifically for writing tasks. It consequently remains an open question how the different interaction modes influence fMRI of specific pen-and-paper NP tests, each test with its associated task demands. Furthermore, fMRI group results have yet to be obtained for tablet-based performance of a task that closely approximates the actual TMT in either interaction mode, nor have such data been studied in relation to actual TMT performance. The present work addresses these knowledge gaps by characterizing tablet-based TMT behavior and brain activity for two groups of young healthy adults, one group performing the TMT with VFHP and one group without VFHP, and with both groups performing the actual TMT a substantial period of time later. It is hypothesized that there are systematic differences in behavior and brain activity between the two groups, which are particularly evident when comparing TMT-B vs. TMT-A. In addition, it is hypothesized that tablet-based TMT performance during fMRI in both interaction modes correlates with actual TMT performance outside the magnet.

## Materials and methods

### Subjects

The study was conducted with the approval of the Research Ethics Board at Sunnybrook Health Sciences Center in Toronto, and with the free and informed consent of the volunteer subjects. All subjects were right-handed as assessed by the Edinburgh Handedness Inventory (Oldfield, 1971); native English speakers; free from standard MRI exclusion criteria (e.g., claustrophobia, ferromagnetic implants); free from any past or present neurological or psychiatric impairments; and recruited from the population of graduate students at the University of Toronto.

Twenty-two young healthy adults performed the experiment (10 male, 12 female; mean age 24.6 ± 3.4). The subjects were randomly assigned to one of two groups, with 11 performing the TMT with VFHP, and the other 11 performing the TMT without VFHP. Both groups were matched in age and sex when appropriate student *t-*tests were performed. In addition, all subjects subsequently performed the standard paper version of the TMT in a separate session under supervision by the same trained test administrator (M.K.). The time interval between TMT fMRI and the standard TMT was ~2 years for each subject, thus removing the confounds of learning and practice on performance of the paper version of the test (Stuss et al., 1987; Basso et al., 1999).

### TMT fMRI design

**Figure 1:**
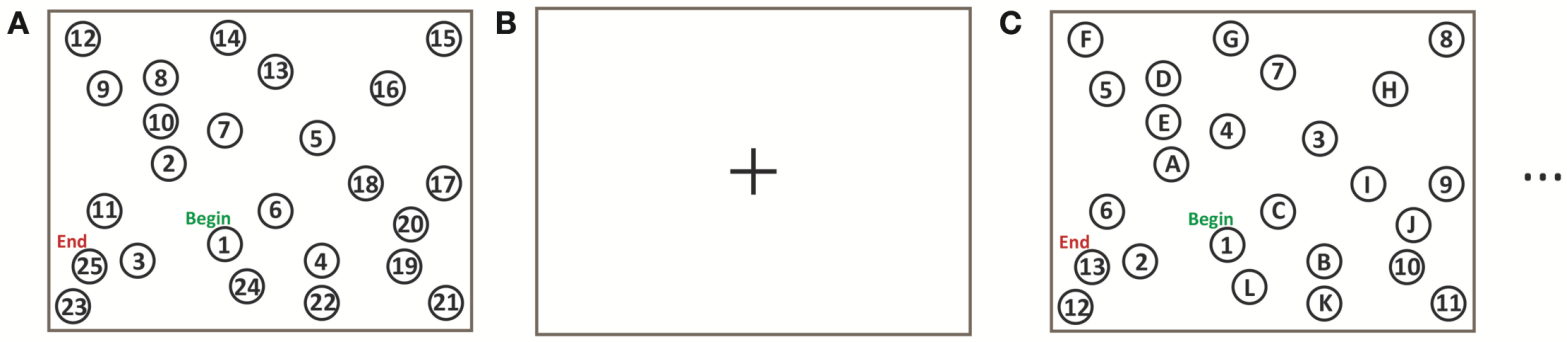
Trail making test (TMT) task design for fMRI experiments: **(A)** TMT-A for 60 s; **(B)** Baseline visual fixation for 10 s; **(C)** TMT-B for 60 s.

The TMT was implemented for fMRI experiments (Figure 1) as described in the introduction (Reitan, 1971; Corrigan and Hinkeldey, 1987; Gaudino et al., 1995; Lezak et al., 2004). The TMT-A task involved the numbers 1–25 pseudo-randomly distributed on the screen. The pseudo-random layout was based on the standard TMT except that the layout was rotated 90° to fit the display. TMT-B involved the numbers 1–13 and the letters A–L in analogous fashion. Subjects were instructed to draw a continuous line (i.e., without lifting their hand) using a stylus to link numbers, or numbers and letters, as required, beginning at the circle marked “Begin” and finishing at the circle marked “End”, as quickly as possible while maintaining accuracy. Task blocks of TMT-A and TMT-B (each of 60 s duration) were repeated in four trials, using a different spatial pattern of numbers/letters in each block. Each TMT block was separated from the next by a baseline condition of visual fixation consisting of a white screen with a central black fixation cross, of 10 s duration. The task design was implemented using a custom program written using E-prime Software (version 2; Psychology Software Tools, Inc., Sharpsburg, PA) that received and interpreted the touch position information from the tablet, and provided task-related feedback in the form of computer graphics superimposed on visual stimuli.

Subjects received training on using the tablet prior to performing the TMT tasks. This involved practicing one sample of each trial, TMT-A and TMT-B, outside the MRI system using the tablet. The fMRI time series data were subsequently collected in one “run” of 9 min and 30 s duration, containing all TMT and baseline blocks (eight stimulus periods and eight baseline periods). The time series included an additional 10 s of dead time at the onset to ensure that the fMRI signal was at equilibrium prior to presenting the first TMT block.

During fMRI, behavioral performance was recorded as a function of time in the form of (x, y) coordinates from the touch-sensitive tablet surface, and contact force from a sensor located at the stylus tip. Both parameters were sampled and logged to a computer file at a rate of ~40 Hz using E-prime software (Psychology Software Tools, Inc., Sharpsburg, PA).

Regarding administration of the standard pen-and-paper test (Reitan, 1971; Corrigan and Hinkeldey, 1987; Gaudino et al., 1995; Lezak et al., 2004), subjects were first given a copy of the TMT-A worksheet and a pen. The test administrator demonstrated the test to the subject using the sample sheet “Trail Making Part A SAMPLE.” Subjects were then instructed to link items on the TMT-A worksheet as quickly as possible while maintaining accuracy, without lifting the pen from the paper. The time to completion was recorded. If the subject made an error, the administrator notified the subject immediately and allowed the subject to correct it. This procedure was repeated for TMT-B.

### fMRI-compatible tablet technology

**Figure 2:**
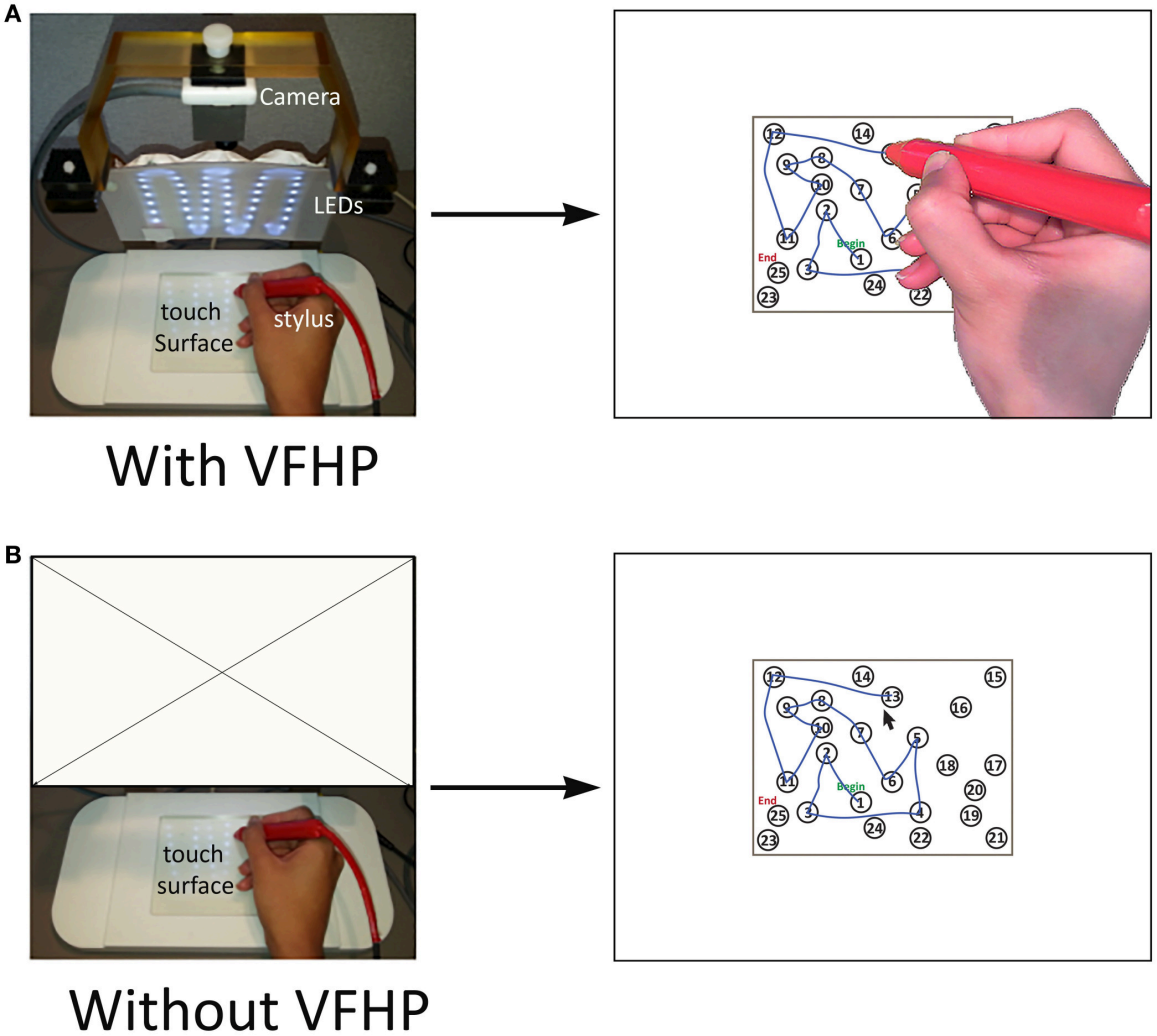
The two modes of interacting with the fMRI-compatible tablet system. Subject performing TMT-A **(A)** with VFHP; **(B)** without VFHP. VFHP, Visual feedback of hand position; LEDs, Light emitting diodes.

The fMRI-compatible tablet system and the two different modes of user interaction are shown in Figure 2. The system included the same resistive touch-sensitive surface used in previous fMRI studies for converting localized contact force to position coordinate values, and for locating these values on a computer display (Tam et al., 2011). In addition, the stylus used for tablet interactions included a force sensor (FSR 400, 30-49649, Interlink Electronics, Camarillo, CA) located at the stylus tip.

For subjects who performed the TMT task design with VFHP (Figure 2A), the tablet was configured with an MRI-compatible video camera with a color CMOS sensor (12M-i, MRC Instruments Gmbh, Germany) and custom light emitting diode (LED) illuminator. A complete description of the fMRI compatible setup is provided in Karimpoor et al. (2015). Using custom programs written in MATLAB (The Mathworks Inc., Natick, MA) a real-time segmentation procedure was used to isolate the hand and stylus from each camera video frame, using a simple skin color detection algorithm performed in Red-Green-Blue (RGB) space (Kovac et al., 2003). In addition, the color properties of the stylus were also adjusted to fall within the RGB distribution of skin color, using a red plastic cover. For this tablet interaction mode, touching the stylus to the tablet and pressing downwards beyond a preconfigured force threshold resulted in “ink” marks at the analogous locations on the display. The camera and stimulus/response video signals were then superimposed in an augmented reality display viewed by the subject.

For subjects who performed “without VFHP”, the tablet was configured to operate with the video camera disconnected (Figure 2B). In this interaction mode, touching the stylus to the tablet resulted in a cursor appearing at the analogous location on the display. Pushing harder with the stylus, beyond a preconfigured force threshold, resulted in “ink” marks at the cursor location.

### fMRI parameters

All imaging was conducted at 3.0 T using a research-dedicated whole-body MRI system (MR750, GE Healthcare, Waukesha, WI), with a standard eight-channel head coil receiver. An angled mirror was attached to the head coil so that the subject could view visual stimuli on a rear-projection screen mounted at the rear of the magnet bore. Anatomical MRI was undertaken using standard inversion recovery-prepped three dimensional (3D) fast spoiled gradient echo imaging (3D FSPGR, inversion time (TI) 300 ms, repetition time (TR) 7.0 ms, echo time (TE) 3.1 ms, flip angle 15°, field of view (FOV) 22 × 22 cm, matrix = 256 × 192, number of slices = 190, slice thickness = 1 mm). These images subsequently served as an anatomical underlay to the color maps of brain activity generated from fMRI data. Functional MRI (fMRI) was undertaken using a T2^*^-weighted spiral in/out pulse sequence (Glover and Law, 2001) to record brain activity via the blood oxygenation level dependent (BOLD) effect (Ogawa et al., 1990, 1992) (TR = 2 s, TE = 30 ms, flip angle 70°, FOV = 20 × 20 cm, matrix = 64 × 64, number of slices = 30, slice thickness = 4.5 mm, voxel size = 3 × 3 × 4.5 mm). Cardiac and respiratory signals were measured during fMRI using a photoplethysmograph attached to the finger of the left hand and a respiratory belt strapped around the torso, respectively.

### Behavioral data analysis

Performance of the standard TMT is typically quantified by measuring the completion times for parts A and B. A slightly different approach was taken during fMRI, however, for two reasons: (1) task blocks were of fixed duration; and (2) the tablet technology permitted more detailed digitized recording of TMT responses. Various metrics were calculated as outlined below, and statistically analyzed from the log file recorded during tablet interactions using custom MATLAB programs.

It was anticipated that some subjects would not complete a given TMT trial within the 60 s block duration, potentially introducing a “ceiling effect” into the completion time data. To account for this possibility, each trial was also scored by determining the number of links completed in a given time (the completion time or the block duration, as appropriate) and then dividing the time by the number of links to estimate the average time to perform each link. The seconds per link quantity is subsequently referred to as Spl. In addition, based on close inspection of tablet performance, subjects often showed some latency period while they performed a visual search prior to executing each linkage. This behavior was quantified as the time duration with stylus movement speed <0.1 pixel/ms at the onset of link performance, averaged over all links completed for TMT-A or TMT-B. This “dwell time” is subsequently referred to as DT. The link length L was also quantified as the average number of pixels that the subject “inked” between two items. Lastly, the stylus contact force F was calculated from the time-averaged force sensor data across trials. In preliminary analysis, it was observed that there was little variation in the force sensor data across TMT-A and TMT-B. Therefore, the F metric was time averaged across all TMT trials for each subject.

All of tablet-based data were assessed for normality using the Shapiro-Wilk test. Differences in the completion time, Spl, DT, and L metrics were investigated using a three-way mixed-effects Analysis of Variance (ANOVA), with tablet mode (with VFHP, without VFHP), and TMT part (TMT-A, TMT-B) as fixed effects, and subjects as random effects. A Bonferroni correction was performed for multiple comparisons. The potential difference in the F metric between the two tablet modes was assessed statistically using the Wilcoxon signed-rank test.

The relationship between tablet-based TMT performance and performance of the standard paper-based TMT was also investigated. First, the standard paper-based TMT scores were computed: completion time for TMT-A (“A”), completion time for TMT-B (“B”), the difference between the completion times for TMT-A and TMT-B (“B-A”), and the ratio score of the TMT-B completion time divided by TMT-A completion time (“B/A”). A paired Student's *t*-test was used to assess whether within-group differences in the B and A values were statistically significant. (The B-A and B/A scores were not manipulated further, but were evaluated subsequently in relation to published normative data). Next, Spl values were calculated from the standard TMT scores by dividing A and B by the number of links in part A and part B, respectively. The difference in Spl parameters for tablet-based TMT performance and standard TMT performance were then assessed using the paired Student's *t*-test. Lastly, the Pearson correlation coefficient, r, was computed to assess the convergent validity between Spl values for standard TMT performance and TMT performance with each tablet mode, for parts A and B.

### fMRI

The map of brain activity associated with performing TMT-B in relation to TMT-A is an example of a “weak contrast” investigating higher-level cognitive effects, rather than a “strong contrast” involving substantial changes in brain activity, such as observed for example in a block design involving a simple motor task vs. rest. Previously, it has been demonstrated that (1) the choices made in fMRI pre-processing pipelines (the procedures conducted to de-noise fMRI data prior to generating activation maps) significantly affect the reliability of extracted brain maps, at both the individual and group level; and (2) weaker task contrasts are more sensitive to these choices, and show a greater benefit from optimized preprocessing strategies (Churchill et al., 2012b). For these reasons, the Optimization of Preprocessing Pipelines for NeuroImaging (OPPNI) software package was used in the present work to optimize the reproducibility of activation maps (Churchill et al., 2012a,b, 2015), rather than applying a fixed generic pre-processing pipeline on all subjects. Within OPPNI, pre-processing pipelines were optimized for each single subject using the NPAIRS method described below (Strother et al., 2002), thereby generating the most reliable and task-predictive activation map for each subject, prior to group analysis.

For each subject, the fMRI time series data were divided into two “split-halves”: split-half one consisted of the first and the second trials of TMT-A and TMT-B, and four baseline blocks; split-half two consisted of the third and fourth trials of TMT-A and TMT-B and another four baseline blocks. The first two time points from each block were discarded to avoid fMRI signal transients and to model stabilized BOLD hemodynamic responses. The fastest time for a subject to complete one block of the TMT-A was 20 s. For consistency, and to eliminate the effects of variable task completion times, only the first 20 s of each block were analyzed for all subjects.

The optimal preprocessing pipelines were selected by computing metrics of prediction (P) and reliability (R) for all preprocessing pipelines, and selecting the pipeline minimizing the Euclidean distance D(P, R) relative to perfect model performance (*P* = 1, *R* = 1). The *P* values were computed using a classifier model based on a single split-half, and measuring the ability to predict experimental conditions in the other split-half using Bayesian posterior probability. The *R* values were computed from the Pearson correlation between split-half activation maps. Pipeline optimization was conducted for each subject, by measuring D(P, R) for all possible combinations of the following preprocessing algorithms, implemented using calls to Analysis of Functional NeuroImages freeware (AFNI) (Cox, 1996) or directly in the OPPNI software. These algorithms were applied in the following fixed order: rigid-body motion correction using *3dvolreg*; cardiac and respiratory physiological noise correction (Glover et al., 2000) using *3dretroicor*; slice timing correction using *3dTshift*; spatial smoothing using a 6 mm full width half maximum (FWHM) Gaussian filter using *3dmerge*; temporal detrending with 0th–3rd order Legendre polynomials using *3dDetrend*; motion covariate regression using Principal Component Analysis (PCA) of *3dvolreg* motion parameter estimates and regressing PCs that accounted for >85% of motion variance; task paradigm covariate regression to protect against over-regression of task-related BOLD signals, using the SPMG1 function (Rasmussen et al., 2012); and global signal regression by removing the first component of PCA (Carbonell et al., 2011). Brain masks were generated using the Oxford Center for fMRI of the Brain (FMRIB) Software Library (FSL) Brain Extraction Tool (Smith et al., 2004).

The individual subject datasets were analyzed using Linear Discriminant Analysis (LDA), a predictive multivariate model, regularized by projecting onto a PCA basis prior to analysis [where the optimal number of PCs was chosen to minimize D(P, R)]. The LDA-PCA algorithm was chosen as a simple, robust classifier model, which allows us to compute (P,R) metrics for each pipeline. In addition, recent work has shown that compared to other multivariate linear classifiers, LDA-PCA exhibits relatively high prediction accuracy and highly reproducible brain maps (Pereira et al., 2009; Misaki et al., 2010; Churchill et al., 2014; Yourganov et al., 2014). For each subject, the brain maps generated by LDA-PCA were converted into Z-scored reproducible statistical parametric maps (rSPMZs) using the procedure described in Strother et al. (2002), prior to group-level analysis. For each subject, their rSPMZ was then transformed into a standard brain atlas space (MNI 152, Mazziotta et al., 2001) as follows: FSL *flirt* was used to compute the rigid-body (6-parameter) transform from EPI to T1 image, and the affine (12-parameter) transform from T1 to MNI template. The net transform matrix from EPI to MNI was then computed, and applied to the rSPMZ.

Group-level analysis was performed by applying PCA to the set of 11 rSPMZs for each group (with/without VFHP). The extracted spatial PCs of each analysis were the multivariate brain patterns that explained the most variance within each group. This was also done in a split-half cross-validation framework as in (Churchill et al., 2015) with 100 resampling iterations, in order to obtain *Z*-scored voxel values for each PC. The brain maps were then thresholded at False-Discovery Rate (FDR) *q* = 0.05 to correct for multiple comparisons (Genovese et al., 2002), and the brain maps were compared between groups.

This analysis approach was developed primarily to generate maps of brain activity for TMT-B vs. TMT-A. Given that extensive areas of brain activity were observed commonly across both tablet interaction modes for each TMT part, however, the average maps for TMT-A vs. baseline and TMT-B vs. baseline were also calculated and reported. Voxels or groups of voxels which passed FDR thresholds of *q* = 0.05 for both TMT-A with VFHP and TMT-A without VFHP were determined using conjunction analysis, providing a mask of commonly activated brain regions. The Mean activation map of TMT-A was calculated across the two modes of tablet interaction using the AFNI function *3dMean* within areas of commonly activated brain regions. The 3dclust command in AFNI (peak activation; cluster threshold = 20 voxels) was used to extract peak coordinates for clusters residing within the mean activation map of TMT-A. Similar steps were taken to provide peak coordinates of commonly activated brain regions for TMT-B. In cases where multiple contiguous clusters were observed (e.g., in frontal, parietal and occipital areas) the spatial coordinates of local extrema were reported briefly, as reasonably appropriate.

Head motion parameters were estimated using rigid body image registration (using the 3dvolreg function in AFNI) implemented in very early steps of the pre-processing pipelines. Temporal standard deviations of each motion parameter (Std) were calculated for each subject in Matlab (the MathWorks Inc.). Differences in Std values for the “with VFHP” and “without VFHP” tablet modes were then assessed statistically across the two subject groups (with/without VFHP) using the Wilcoxon signed-rank test.

## Results

### TMT performance

After training outside the MRI system, all subjects were able to perform TMT with the tablet successfully during fMRI. Subjects who performed without VFHP had completion times (group mean with standard deviation shown in brackets) of 39.7 (11.7) s and 48.9 (11.1) s for TMT-A and TMT-B, respectively; those who performed with VFHP had analogous values of 46.2 (11.3) and 53.2 (9.8) s. As suggested by these values and as mentioned above, numerous subjects were unable to complete all linkages within each block duration of 60 s. For example, seven subjects in each group failed to complete at least one trial of TMT-B in this manner. Performance of the Shapiro-Wilk test indicated that the completion times for both groups were not normally distributed. Preliminary 3-way ANOVA results suggested a statistically significant main effect of TMT part for both interaction modes (with TMT-B showing increased completion time) and no interaction effects between modes. A Wilcoxon signed-rank test was also conducted between completion times for TMT with VFHP (B-A) and without VFHP (B-A), and in an analogous manner for the B/A ratio. There were no statistically significant differences observed in either case.

**Figure 3:**
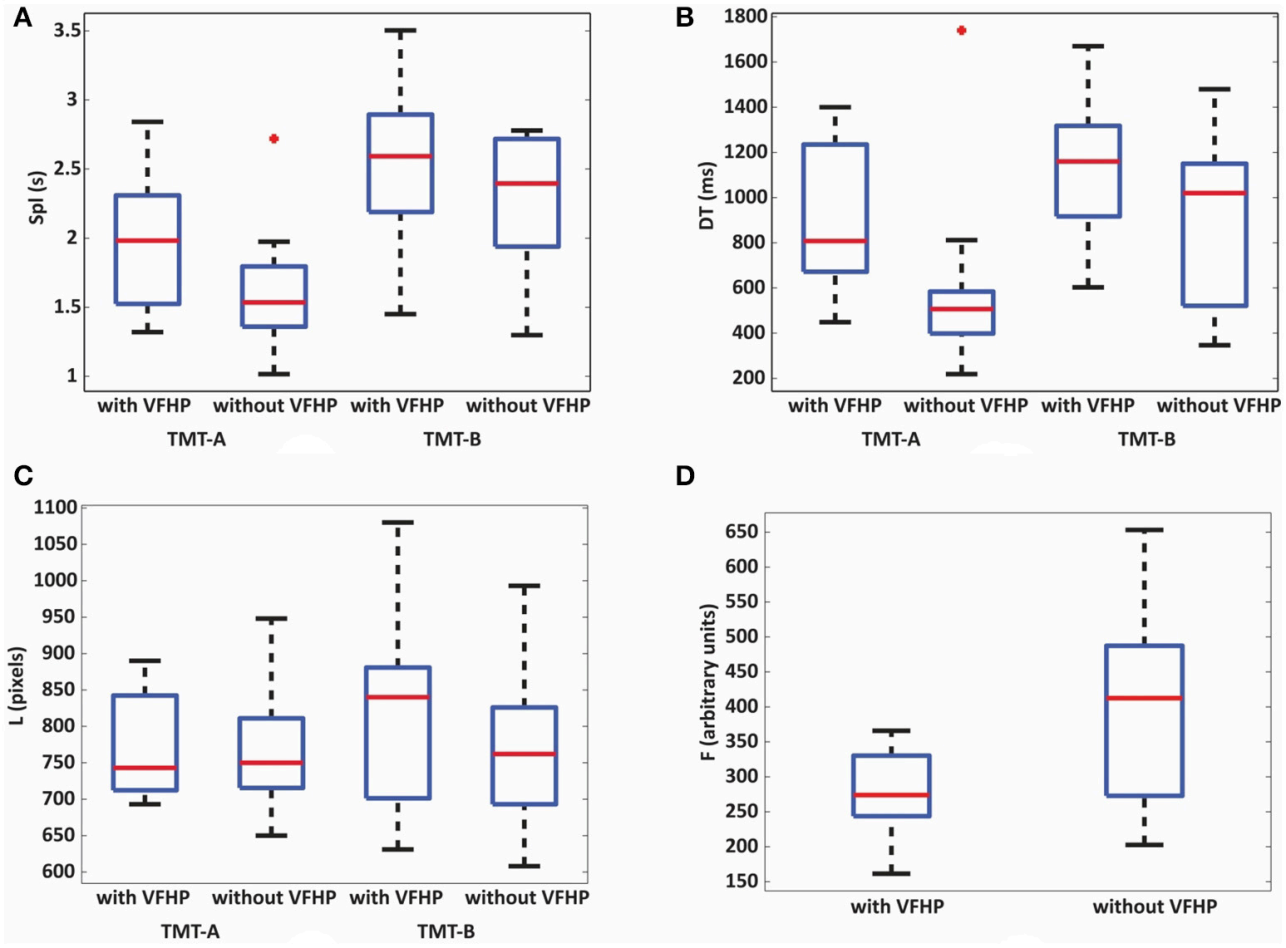
Box and whisker plots of behavioral performance parameters for subjects who performed all TMT tasks either with VFHP or without VFHP: **(A)** seconds per link (Spl); **(B)** dwell time (DT); **(C)** linkage length (L); **(D)** contact force (F). For each box, the center horizontal line shows the median parameter value, and the edges of the box are estimates of the first and third quartile. The whiskers extend to the most extreme data points not considered outliers (2.7 times the sample standard deviation assuming a normal distribution). Outlier data points are shown as crosses.

Given the above mentioned ceiling effects, behavioral analysis subsequently focused on the results for the additional behavioral metrics developed specifically for the tablet (Spl, DT, L, and F) which were all found to be normally distributed based on Shapiro-Wilk tests, except for DT metric of TMT-B in the tablet mode without VFHP. Figure 3 shows box and whisker plots depicting the median and interquartile range (IQR) for each metric, with the box bounding the first and third quartiles, and the whiskers extending to the most extreme data points not considered outliers (2.7 times the sample standard deviation). Outlier data points are shown as crosses. In general, performance differences were minor between the two tablet modes. The only statistically significant ANOVA result for time-related performance measures was a main effect of TMT part (*p* < 0.05, Bonferroni corrected) with increased Spl (Figure 3A) and DT (Figure 3B) for TMT-B compared to TMT-A, across both tablet modes. Furthermore, after performing *post-hoc t*-tests, performance of TMT-A with VFHP showed a non-significant but strong trend of increased DT (*p* < 0.04, uncorrected) compared to performance without VFHP. The analogous effect was not observed for TMT-B. No statistically significant results were observed in the linkage length L across TMT-A and TMT-B or the two tablet modes (Figure 3C). However, TMT performance with VFHP showed decreased *F* values compared to performance without VFHP, during the first 20 s of each block included in the analysis (Figure 3D; *p* < 0.05, corrected).

Table 1 lists the scores for all subjects performing the standard TMT, including Spl values for TMT-A and TMT-B. As expected, the group mean completion time for TMT-B was significantly higher than for TMT-A (*p* < 0.001, corrected). The performance metrics (A, B, B-A, B/A) also varied considerably across the group but are consistent with published normative data for this age range and education level (Giovagnoli et al., 1996; Tombaugh, 2004). It is evident from Table 1, Figure 3A that the tablet-based Spl values are somewhat elevated compared to the analogous values for the standard TMT. This was also quantitatively measured by performing a paired *t*-test, which showed significantly increased Spl values for tablet-based TMT performance compared to the standard TMT performance (*p* < 0.001 for both parts of the TMT considered separately). In addition, tablet-based Spl values were significantly correlated to pen-and-paper Spl values for TMT-A for both tablet modes (with VFHP: Pearson correlation coefficient 0.64, *p* < 0.05; without VFHP: Pearson correlation coefficient 0.72, *p* < 0.05). For TMT-B, significant correlations in Spl values were not observed.

**Table 1:** Scores for subjects (*N* = 22) performing the standard TMT.

	**Mean**	**SD**	**Range**
A	20.3	6.2	25.1
B	42.3	14.2	57.3
B–A	22.0	13.9	66.3
B/A	2.19	0.80	3.12
Spl (TMT-A)	0.85	0.26	1.04
Spl (TMT-B)	1.84	0.62	2.49

SD, Standard deviation.

### Brain activity

**Figure 4:**
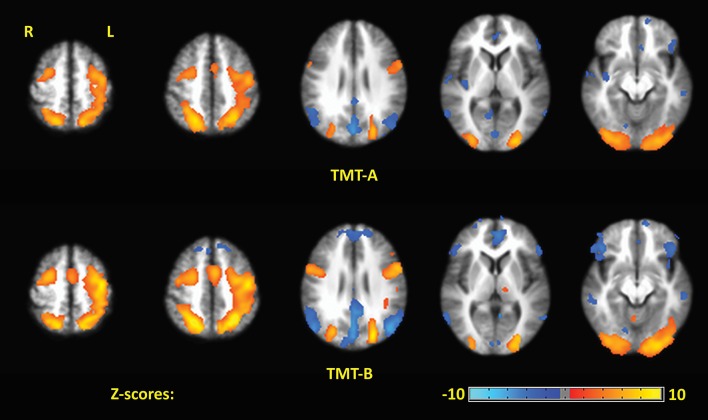
The first principal component (PC1) depicting brain activity for commonly activated brain regions in TMT-A vs. baseline and TMT-B vs. baseline for the two tablet interaction modes. Activation maps are thresholded at a false discovery rate of *q* = 0.05.

Figure 4 shows the Z-scored maps for the first principal component (PC1), reflecting the most common pattern of brain activity for TMT-A vs. baseline and TMT-B vs. baseline, for selected slice locations. Maps for PC2 are not shown due to lack of significant voxel values after FDR correction, for either parts of the TMT performed with VFHP, very sparse results for either part performed without VFHP, and thus zero conjunction between the interaction modes. The complete sets of regions commonly activated across tablet modes for TMT-A vs. baseline and TMT-B vs. baseline are listed in Tables 2, 3, respectively, providing the location and value of the maximum Z score. Commonly activated brain regions for TMT-A vs. baseline include bilateral superior parietal lobule (SPL), inferior parietal lobule (IPL), inferior frontal gyrus (IFG), supplementary motor area (SMA), medial frontal gyrus (MeFG), premotor region of the middle frontal gyrus (MiFG), middle temporal gyrus (MiTG), visual and visual association areas; and left lateralized pre-central and post-central gyri. Commonly activated regions for TMT-B vs. baseline also involve the bilateral SPL, IPL, MiFG premotor regions, MiTG, SMA, left lateralized pre-central and post central gyri, and primary visual and visual association areas. In qualitative comparison to TMT-A vs. baseline, slightly more extensive activity is observed in the IFG, premotor area of MiFG, SPL, IPL, pre-central gyrus, visual association areas, MiTG, with recruitment of additional brain regions such as the left DLPFC, and left MiFG.

**Table 2:** Commonly activated brain regions identified for TMT-A vs. baseline in MNI coordinate space across both tablet modes.

**Active region**	**Hemisphere**	**Z–score**	**MNI Coordinates (mm)**		
SPL	R	9.1	20	−66	54
SPL	L	8.2	−26	−64	54
MiOG	L	7.9	−26	−92	4
MiOG	R	6.6	28	−92	4
Precentral Gyrus	L	6.8	−44	−16	56
IPL	L	6.9	−30	−58	48
IPL	R	5.4	30	−50	46
MiTG	L	6.4	−28	−76	18
MiTG	R	4.7	30	−78	22
MiFG	L	5.7	−36	−6	60
MiFG	R	4.9	24	−4	60
Cuneus	L	5.3	−16	−98	−8
Cuneus	R	4	14	−98	−8
IFG	L	4.7	−60	8	26
IFG	R	4.3	44	4	26
SMA/MeFG	L	4.3	−4	2	52
Post Central Gyrus	L	3.6	−40	−28	54
Precuneus	L	−6.5	0	−78	34
MiTG	L	−4.1	−60	−28	−14
Insula	R	−4.1	40	−18	12
STG	R	−4	58	−54	14
IFG	R	−3.9	54	18	−4
IFG	L	−3.6	−56	32	2
PC	R	−3.6	8	−60	10
MiTG	R	−3.3	60	−32	−6
AC	L	−3.5	−6	28	−6
Parahippocampal gyrus	R	−3.2	34	−10	−26
AC	R	–3.1	0	42	6

*IFG, MiFG, MeFG, inferior, middle, medial frontal gyrus; STG, MiTG, superior, middle temporal gyrus; MiOG, middle occipital gyrus; IPL, SPL, inferior, superior parietal lobule; AC, anterior cingulate; PC, posterior cingulate; SMA, supplementary motor area; MNI, Montreal Neurological Institute*.

**Table 3:** Commonly activated brain regions identified for TMT-B vs. baseline in MNI coordinate space across both tablet modes.

**Active Region**	**Hemisphere**	**Z–score**	**MNI Coordinates (mm)**		
IPL	L	10.7	−30	−54	48
SPL	R	10.5	20	−64	54
SPL	L	9.6	−28	−62	52
Pre-central gyrus	L	10.2	−42	−16	58
MiTG	L	8.381	−32	−78	18
MiTG	R	7.4	30	−78	18
Lingual gyrus	L	8	−24	−92	−8
Lingual gyrus	R	5.8	20	−22	−8
Post-central gyrus	L	7.9	−42	−20	54
MiFG	R	7.2	26	−4	58
MiFG	L	7.6	−34	−6	60
SMA/MeFG	L	7	−4	2	52
IOG	L	7.6	−32	−90	−12
IOG	R	6.6	36	−86	−10
Declive of Vermis	R	6.5	4	−70	−20
IFG	R	6.3	44	2	26
IFG	L	3.5	−48	24	24
MiOG	R	4.3	30	−92	2
DLPC/MiFG	L	3.4	−44	38	22
Angular gyrus/MiTG	L	−6.5	−46	−70	30
Angular gyrus/MiTG	R	−5.9	52	−70	26
Pre-cuneus	L	−6.1	0	−70	28
PC	L	−3.7	−6	−58	2
SFG	L	−5.1	−18	42	44
IFG	R	−5.1	44	32	−12
IFG	L	−4.8	−44	28	−16
AC	L	−4.5	−4	48	2
MiTG	L	−4.2	−60	−26	−14
Insula	R	−3.5	40	−18	12

*IFG, MiFG, MeFG, inferior, middle, medial frontal gyrus; DLPC, dorsolateral prefrontal cortex; MiTG, middle temporal gyrus; IOG, MiOG, inferior, middle occipital gyrus; SPL, superior parietal lobule; AC, anterior cingulate; PC, posterior cingulate; SMA, supplementary motor area; MNI, Montreal Neurological Institute*.

No significant differences in overall motion parameters were observed between the two tablet interaction modes. Some differences in brain activity were also observed qualitatively between the two tablet modes. The extent of activity was larger within multiple regions for subjects who performed TMT-A and TMT-B without VFHP than for subjects who performed the tasks with VFHP. For example, the former group showed more extended bilateral involvement of the occipital and parietal lobes. In addition, subjects who performed the TMT with VFHP showed more left-lateralized activation of sensorimotor regions of the brain during TMT-B and TMT-A compared to subjects who performed without VFHP. It was also evident that the activation maps for both TMT parts were slightly less overlapped for subjects who performed with VFHP than for those who performed without VFHP (Jaccard indices of 0.58 vs. 0.62, respectively).

**Figure 5:**
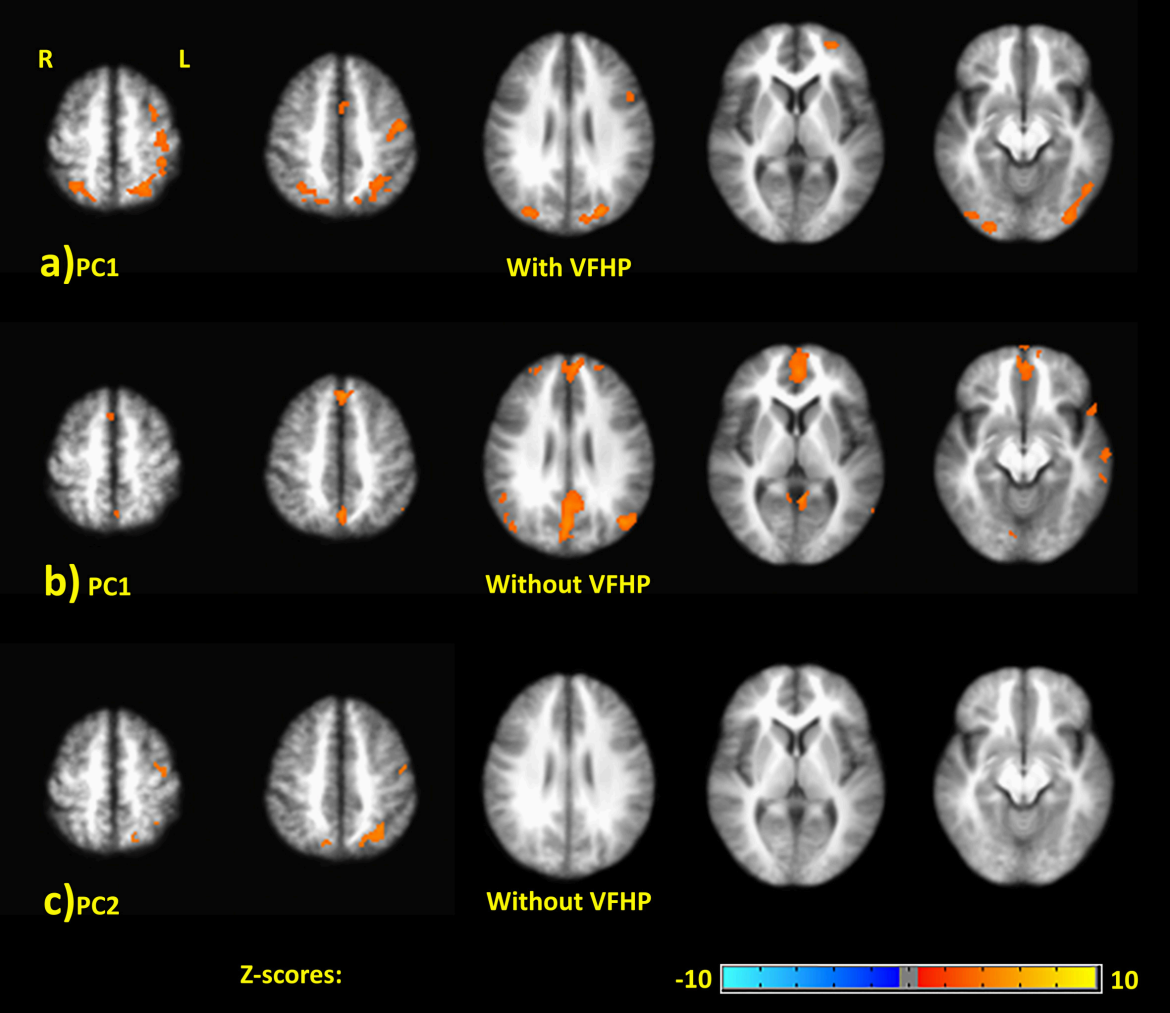
Principal components (PCs) depicting brain activity for TMT-B vs. TMT-A. **(A)** PC1 activation map for performance with VFHP. **(B)** PC1 activation map for performance without VFHP. **(C)** PC2 activation map for performance without VFHP. Activation maps are thresholded at a false discovery rate of *q* = 0.05. The PC2 activation maps for performance with VFHP are not statistically significant.

Figure 5 shows representative activation maps of PCs for TMT-B vs. TMT-A for the two tablet modes of interaction. Activation details for TMT-B vs. TMT-A are also listed in Table 4 (performance with VFHP) and Table 5 (performance without VFHP). Subjects who performed the TMT with VFHP showed only a single significant PC1 (Figure 5a). Activation was left-lateralized in the DLPFC, IFG, MiFG, pre-central and post-central gyrus; and bi-lateral in the MeFG, SPL, precuneus, cuneus, and cingulate gyrus. Subjects who performed the TMT without VFHP showed activation for TMT-B vs. TMT-A, with two significant PCs (Figures 5b,c). Activated regions in PC1 included superior frontal, anterior cingulate (AC), MiFG, MeFG, IPL and anterior precuneus (aPCu); as well as the bilateral precuneus, and posterior cingulate (PC). Activated regions in PC2 showed some commonality to those shown for PC1 with VFHP, but with reduced extent of significant voxels.

**Table 4:** Brain regions identified for the contrast of TMT-B vs. TMT-A, PC1, in MNI space for tablet interactions with VFHP.

**Active region**	**Hemisphere**	**Z–score**	**MNI coordinates (mm)**		
Declive	L	5.2	−4	−80	−24
Pre-central gyrus	L	5.2	−46	−12	54
SPL	R	5.1	32	−60	56
SOG	L	5	−28	−78	24
SPL	L	4.6	−26	−58	60
MiTG	L	4.6	−54	−58	−14
Pre-cuneus	R	4.4	28	−80	34
DLPFC/MiFG	L	4.2	−30	54	6
MiFG/premotor	L	4	−34	−2	58
Declive	R	4	46	−72	−30
Cingulate gyrus	L	3.8	−2	6	48
IOG	R	3.7	40	−82	−10
IFG	L	3.6	−50	12	30

*IFG, MiFG, inferior, middle frontal gyrus; DLPFC, dorsolateral prefrontal cortex; MiTG, middle temporal gyrus; IOG, SOG, inferior, superior occipital gyrus; SPL, superior parietal lobule; MNI, Montreal Neurological Institute*.

**Table 5:** Brain regions identified for the contrast of TMT-B vs. TMT-A, PC1 and PC2, in MNI space for tablet interactions without VFHP.

**Active region (PC1)**	**Hemisphere**	**Z–score**	**MNI coordinates (mm)**		
Pre-cuneus	L	6.3	−2	−56	38
MeFG	L	5.9	0	56	−4
STG	L	5.8	−62	−58	12
SFG	L	5.4	−2	30	54
AC	L	4.8	−4	48	8
Culmen	R	4.7	8	−46	0
SFG	R	4.7	20	64	14
MiFG	R	4.5	40	32	42
Post-central gyrus	L	4.4	−6	−46	70
Posterior cingulate	L	4.4	−0	−46	22
MeFG	R	4.4	6	50	40
IFG	L	4.3	−52	24	−16
MiFG/ premotor	L	3.8	−50	12	46
STG	R	3.7	56	−64	22
IPL	L	3.7	−58	−56	40
Lingual gyrus	R	3.7	10	−78	−14
Supramarginal gyrus	R	3.6	50	−50	30
Supramarginal gyrus	L	3.6	−66	−46	28
Precuneus/aPCu	L	3.1	−4	−62	60
**Active Region (PC2)**	**Hemisphere**	**Z–score**	**MNI coordinates (mm)**		
SPL	L	4.8	−32	−58	52
Pre-central gyrus	L	4.3	−48	−12	54
Pre-cuneus	R	4.3	12	−70	52

*AC, anterior cingulate; aPCu, anterior pre-cuneus; IFG, MiFG, MeFG, inferior, middle, medial frontal gyrus; PC, posterior cinulate; STG, superior temporal gyrus; IPL, SPL, inferior, superior parietal lobule; MNI, Montreal Neurological Institute*.

## Discussion

The present work constitutes ongoing validation of this fMRI-compatible tablet technology, and its application to depict the brain activity that supports performance of NP tests involving pen-and-paper responses. Specifically, a tablet-based version of the TMT was implemented and fMRI of young healthy adults was undertaken to characterize brain activity in both parts of the test, and to study the effect of the two different tablet modes of interaction (with VFHP and without VFHP). The work is important to inform the design of future fMRI studies involving the TMT and the tablet in patient populations, as well as application of the tablet technology with other NP tests. This work also investigates the tradeoffs of using “augmented reality” technology when investigating brain function associated with NP tests. The following discussion describes the ramifications of the study, focusing first on the behavioral performance aspects and then the brain activity that was observed when the TMT was administered in the two different interaction modes.

### Behavior

The behavioral performance results of the study are encouraging, as the tablet-based TMT results in both interaction mode showed reasonable agreement with the standard TMT results, and the performance differences between the two tablet modes were minor. Regarding administration of the standard TMT, subjects were assessed 2 years after participating in the fMRI study such that the influence of tablet testing on pen-and-paper outcomes was negligible. This is supported by the finding that the standard TMT scores for the subjects were consistent with the available normative data (Giovagnoli et al., 1996; Tombaugh, 2004). For TMT-A, despite the Spl values for tablet-based performance being approximately twice that of the pen-and-paper responses, statistically significant *r* values of 0.64 for performance with VFHP and 0.72 without VFHP showed good convergent validity in both cases. For context, these correlation values are similar to the reliability values reported across test-retest studies of the standard TMT. When Levine et al. (2004) retested 1047 healthy adults after an interval of 2–24 months, reliability of 0.7 was measured for both TMT parts whereas Matarazzo et al. (1974) used a 12 week test-retest interval and reported reliability values of 0.46 and 0.44 (TMT-A and TMT-B, respectively) for 29 young, healthy, normal males. Dikmen et al. (1999) measured test-retest reliability in 384 normal healthy adults over 11 months and reported reliability values of 0.79 and 0.89 for TMT-A and TMT-B, respectively. Based on the research conducted here, a logical next step is to conduct test-retest fMRI studies of tablet based TMT performance. This will be important for future clinically-oriented fMRI studies involving patients, because the reliability of fMRI single-subject results is known to be reduced in relation to group results.

As for the standard TMT, the Spl data showed that subjects using the tablet in either interaction mode completed links more slowly when performing TMT-B than when performing TMT-A. In addition, the Spl values for tablet-based TMT-B were only slightly elevated compared to those of the standard TMT. However, the extent of slowing in the standard TMT (~two-fold, as indicated by the B/A scores) was much less for tablet-based TMT primarily because the Spl values for TMT-A were elevated. The *r* values of correlation between tablet-based TMT-B and standard TMT scores were also not statistically significant for either interaction mode. Although this pattern of results is not unreasonable given more variable performance of subjects during TMT-B and low sample size, it is also highly likely that some of the inherent differences in task demands between standard TMT and tablet-based TMT must play a role. Although the tablet technology enables fMRI of writing and drawing tasks, the challenging fMRI environment inevitably necessitates some compromises to task performance, irrespective of the interaction mode. For fMRI signal contrast-to-noise ratio considerations, for example, subjects were required to complete multiple blocks of TMT-A and TMT-B with the tablet with varying stimuli, whereas the standard TMT is administered once. As a consequence, the tablet-based data are potentially confounded by short-term learning effects. There are also some noteworthy differences in sensorimotor task demands when performing the standard TMT compared to tablet-based TMT. In the latter case, subjects must perform tablet interactions while supine and viewing a projection screen, in an environment with considerable acoustic noise. Whereas the standard TMT test is performed on A4 paper, the area of the touch-sensitive surface of the tablet is constrained by the manufacturer and by the confines of the magnet bore. More precise movements are required with the tablet as a result.

The data in Figure 3, Table 1 also indicate that beyond the differences in task demand between the tablet-based TMT and the standard TMT, some subtle differences exist between the two modes of tablet interaction. Interaction with VFHP was shown previously to help subjects locate the stylus tip more effectively and to enable performance with less contact force than when interacting without VFHP during writing tasks, suggesting lower sensorimotor demands (Karimpoor et al., 2015). The latter finding was replicated in the present study. In the standard TMT, however, subjects are instructed to maintain pen-paper contact during task performance, but no instructions are given about coping with how their hand and forearm potentially block their view of the symbol locations. When using the tablet with VFHP, this visual obstruction problem is more pronounced than when performing with pen-and-paper. The tablet surface is viewed from “top-down” perspective with the TMT symbols confined to a smaller area. In comparison, tablet interaction without VFHP enables subjects to locate where to make links using a cursor, without visual obstruction but with elevated proprioceptive demands in the tablet implementation studied here. The visual obstruction effect was apparent for the TMT-A condition, for which subjects performing with VFHP had increased DT values compared to subjects performing without VFHP. The analogous effect was not observed for the TMT-B condition, likely because it was masked by the additional visual search and set-switching aspects of this more demanding task. Interestingly, these slightly different sensorimotor demands for tablet interactions did not ultimately have a major impact on the visible drawing behavior for young healthy adults, as no statistically significant differences in the link length metric L were observed across TMT-A and TMT-B, and no interaction effects for TMT-B vs. TMT-A were observed across the tablet modes.

### Brain activity

To our knowledge, no other fMRI study has been performed that directly attempts to characterize TMT-A and TMT-B brain activity in detail with a realistic response mode, including comparison with performance on the actual paper and pencil test. Brain maps were reported at 1.5 T for TMT-B vs. TMT-A performed with an MRI-compatible fiber-optic writing device (Zakzanis et al., 2005), although device interactions were less intuitive, ecological validity was not quantified and the baseline task condition required subjects to link empty circles in random order. In the present study, conducted at 3.0 T for enhanced fMRI signal sensitivity with a much more intuitive response device, visual fixation was used as the baseline condition to reveal activations in sensorimotor and visual spatial processing areas in detail. The TMT-A vs. baseline, TMT-B vs. baseline and TMT-B vs. TMT-A activation maps are subsequently discussed further below.

Considering TMT-A vs. baseline first, brain activity was observed across both tablet modes that was consistent with performing a sensorimotor task using the dominant hand. Strong activations were observed in left lateralized primary somatosensory and motor cortex (associated with the tactile and proprioceptive sensory inputs used ultimately to direct hand and arm movement), as well as bilateral SMA and premotor areas, extending to the bilateral parietal lobe and IFG, and bilateral primary visual and visual association areas. Activation of pre-central, premotor and SMA regions is consistent with planning stylus movements that are required to complete linkages between successive numbers. Notably, the bilateral premotor activity is consistent with dynamic visuospatial imagery (Richter et al., 2000) which subjects may engage in the process of planning to create each linkage. Strong bilateral activation of the SPL and MiOG is consistent with the mental processing that supports visual search behavior (Leonards et al., 2000; Nobre et al., 2003) which is also a requirement for performing TMT-A successfully (Crowe, 1998; Sánchez-Cubillo et al., 2009).

Similarly, the activations observed for TMT-B vs. baseline in both tablet interaction modes included all the regions described for TMT-A immediately above. However, the observed activations were more extensive and pronounced in the bilateral IFG, bilateral premotor areas, bilateral SPL, left pre-central gyrus, IPL, MiTG, and visual association areas. In addition, multiple new brain regions were engaged, particularly in the left DLPF, and MiFG. Areal expansion of the identified brain regions is consistent with increased sensorimotor and visual-spatial processing demands required for performing TMT-B compared to TMT-A (Heilbronner et al., 1991; Stuss et al., 2001; Moll et al., 2002; Zakzanis et al., 2005; Allen et al., 2011). In addition, the increased bilateral IFG activation is consistent with additional mechanisms required to support TMT-B performance. The IFG is known to be involved in language processing, especially in the left hemisphere (Friederici et al., 2003; Winhuisen et al., 2005), which supports increased language processing demands of TMT-B in relation to TMT-A. The right IFG is also engaged during set-switching (Dreher and Berman, 2002; Moll et al., 2002; Brass et al., 2003), an executive function which is required to perform TMT-B successfully (Arbuthnott and Frank, 2000; Sánchez-Cubillo et al., 2009). The role of left DLPFC in executive function, particularly in refocusing attention during set-switching (Dove et al., 2000) and in perceptual decision-making (Heekeren et al., 2006), is consistent with its observation for TMT-B vs. baseline as reported in previous functional neuroimaging studies of TMT performance (Zakzanis et al., 2005) as well as in studies of TMT sensitivity and specificity involving patients with brain lesions (Stuss et al., 2001).

Two other brain regions that were active during TMT-B vs. baseline are also noteworthy. First, bilateral activity in the IPL was weighted toward the left hemisphere. Previous NP and neuroimaging studies have demonstrated the crucial role of the left IPL in number processing (Chochon et al., 1999; Dehaene et al., 2003). Although TMT-B and TMT-A both require number processing, the TMT-B scenario is more demanding because of the set-switching between number processing and letter processing. This is consistent with stronger activation of left IPL observed in TMT-B vs. baseline compared to TMT-A vs. baseline. Second, activation was strongly increased in MiTG for TMT-B vs. baseline in relation to TMT-A vs. baseline. Neuroimaging studies have shown that the right MiTG is involved in working memory processing during number letter sequencing tasks (Haut et al., 2000) and during verbal and non-verbal semantic memory processing (Visser et al., 2012). The left MiTG has been found to be active during the perception of biological motion such as hand movements (Bonda et al., 1996; Puce et al., 1998), consistent with viewing visual feedback of movements made by the subject on the tablet.

The discussion above pertains to the areas of activation observed for both tablet modes of interaction. Although both tablet modes produced activation patterns that were very similar, some small differences were observed qualitatively. For both TMT-B and TMT-A activation maps, the extent and amplitude of activation was greater for performance without VFHP in relation to performance with VFHP in many regions including the post-central gyrus, anterior pre-cuneus, and primary visual cortex. This is consistent with the notion that more demands are placed on sensorimotor processing when performing without VFHP, to support and maintain adequate motor performance. Another interesting observation was that the use of a baseline task involving visual fixation revealed components of the default mode network (DMN) (Greicius et al., 2003) with negative *Z*-score values in both TMT-B and TMT-A activation maps. However, DMN regions were revealed to a greater extent when performing without VFHP compared to performing with VFHP. As the DMN is engaged not only at wakeful rest, but also when thinking about the past and planning for the future (Greicius and Menon, 2004; Sestieri et al., 2011), the DMN activation differences observed may reflect differences in the mental state of subjects during baseline conditions when faced with somewhat more challenging task demands associated with performing tablet interactions without VFHP in relation to performing with VFHP. Unfortunately, tablet performance and video camera data were not recorded during baseline conditions, and thus group differences cannot be analyzed in the present study. Future research should include such recordings.

Turning to brain activity associated with the weak contrast of TMT-B vs. TMT-A, subjects who performed the TMT with and without VFHP showed very slight differences in the group patterns of distributed brain activity. Subjects who performed with VFHP generated left-lateralized activation patterns in regions such as the DLPFC, MiFG, and IFG, consistent with the increased demands on executive function required for performing TMT-B compared to TMT-A. As mentioned above, activation of left DLPFC has been observed in previous fMRI studies attempting to identify the neural correlates of TMT-B vs. TMT-A (Moll et al., 2002; Zakzanis et al., 2005; Allen et al., 2011; Churchill et al., 2012b, 2015). Activation of left DLPFC during TMT performance has also been observed using functional near-infrared spectroscopy (fNIRS) to measure changes in the concentration of oxygenated and deoxygenated hemoglobin in neurovasculature at the brain surface (Hagen et al., 2014; Müller et al., 2014). Compared to fMRI, however, fNIRS provides lower spatial resolution and lacks the ability to detect brain activity at depth. In addition to executive functioning, activation maps of TMT-B vs. TMT-A for subjects who made tablet interactions with VFHP showed areas involved in motor control, motor planning and visuospatial processing. These findings are expected given that TMT-B performance requires more motor planning and visual search than TMT-A (Lezak et al., 2004). The regions involved in motor control have been discussed above, and are consistent with the previous findings of Zakzanis et al. (2005). The observed activations of the SPL as well as frontal and occipital areas, are consistent with previous literature implicating the role these regions in visual search abilities (Leonards et al., 2000; Nobre et al., 2003). Additional areas of activation included the AC, recognized to play an important role in performance monitoring, response control and error detection during complex and cognitively demanding tasks such as TMT-B (Carter et al., 1998; Brown and Braver, 2005), more so than during TMT-A (Zakzanis et al., 2005; Allen et al., 2011).

Subjects who performed cursor-based interactions without VFHP expressed qualitatively similar activity for TMT-B vs. TMT-A, but not as strongly. Instead, these subjects expressed predominantly medial brain activity, including bilateral SFG, MeFG and the AC, and aPCu in medial parietal cortex. The SFG is involved with higher orders of working memory processing and spatial cognition (Du Boisgueheneuc et al., 2006). The AC is known to be associated with performance monitoring, error detection, feedback, uncertainty, response inhibition (Bush et al., 2000; Hester et al., 2005; Nachev et al., 2008). In particular, the aPCu is involved in proprioception (Filimon et al., 2009). This overall pattern of brain activation is consistent with more demands to monitor performance, detect errors and inhibit responses (Nachev et al., 2008), as well as more reliance on proprioception as needed to locate the stylus tip while performing TMT without VFHP. Quantitative analysis of the mean force data is also consistent with placing more reliance on proprioception and cursor activation during tablet interactions in the absence of VFHP particularly when comparing performance of TMT-B to performance of TMT-A.

As mentioned above, although the differences in brain activation activity for TMT-B vs. TMT-A across the two tablet modes are quite plausible in consideration of previously reported literature, these effects are a subjective interpretation and should be considered trends rather than statistically significant results. For more objective analysis, additional brain mapping was performed to investigate an LDA model on optimized single-subject maps of TMT-B vs. TMT-A for the groups of subjects in the two tablet modes (with VFHP, without VFHP). No brain regions showed statistically significant interaction effects after FDR correction for multiple comparisons. It is possible that statistically significant effects could be observed with a larger sample size, or if the group demographics were changed (for example, to include patients with brain impairments).

Although the TMT test is among the very oldest NP tests used widely around the world, relatively little work has been done in understanding the neural correlates of this test due to challenges involved during functional neuroimaging procedures. We have presented an fMRI-compatible version of the TMT, which is designed to engage all of the primary cognitive components important to this NP test. This study addresses important questions in understanding the underlying processes of the TMT. The traditional picture that the TMT is sensitive primarily to regions of the frontal lobe has to be considered overly simplistic in light of the present fMRI findings and other functional neuroimaging literature on the subject. The complex network of brain regions determined for TMT-B and TMT-A, and the difference in activity between both TMT parts suggests that patients that have lesions in any of these regions or their interconnections may exhibit impaired TMT performance (Future work involving fMRI of patients will obviously be required to verify this prediction).

In addition, our findings show that in healthy young adults, TMT performance and the underlying brain activity are very similar for both interaction modes of the tablet device. In the future, it will be interesting to investigate whether similar findings are observed in patients with various aspects of sensory, cognitive and motor impairment. Notably, the hand obstructs visual stimuli in the VFHP setup and this may have implications for applying this technology to assess aging or patient populations. Based on the above concerns and positive results of the present work, the next potential step will be building a tablet with a bigger active touch area to provide subjects with a bigger FOV and reduce the effects of VFHP obstructing visual stimuli. However, challenges of this approach include inadvertent touches, increased head motion, and increased fatigue. Semi-transparent VFHP or other hand representations, such as an altered viewing angle, could also be used in the future to reduce the obstruction while providing rich hand guidance.

## Author contributions

MK: Designing the entire work, data acquisition and experimental setup, data analysis and interpretation. NC: Contributions to the work conception, data analysis, and interpretation of data. FT: Contributions to the work conception and experimental setup. CF: Contributions to the work conception. TS: Contributions to the work conception and data interpretation. SG: Designing the work, analysis and interpretation of data. All authors: Critically revising the work, final approval of the work, and ensuring questions related to the work accuracy are investigated and resolved.

### Conflict of interest statement

This work has used a technology which was provisionally patented in 2014. Patent title: Systems and Methods for Providing Visual Feedback of Touch Panel Input During Magnetic Resonance Imaging. Publication number: 20160120437. The authors declare that the research was conducted in the absence of any commercial or financial relationships that could be construed as a potential conflict of interest.
